# Endothelium- and Fibroblast-Derived C-Type Natriuretic Peptide Prevents the Development and Progression of Aortic Aneurysm

**DOI:** 10.1161/ATVBAHA.124.322350

**Published:** 2025-04-03

**Authors:** Aisah A. Aubdool, Amie J. Moyes, Cristina Perez-Ternero, Reshma S. Baliga, Jaspinder Singh Sanghera, M. Taaha Syed, Kareemah Jaigirdar, Anmolpreet K. Panesar, Janice C. Tsui, Yanming Li, Hernan G. Vasquez, Ying H. Shen, Scott A. LeMaire, Juliette Raffort, Ziad Mallat, Hong S. Lu, Alan Daugherty, Adrian J. Hobbs

**Affiliations:** 1 William Harvey Research Institute, Faculty of Medicine and Dentistry, Barts & The London Hospitals, Queen Mary University of London, United Kingdom (A.A.A., A.J.M., C.P.-T., R.S.B., J.S.S., M.T.S., K.J., A.K.P., A.J.H.).; 2 Division of Surgery and Interventional Science, University College London and Royal Free London NHS Foundation Trust, United Kingdom (J.C.T.).; 3 Division of Cardiothoracic Surgery, Michael E. DeBakey Department of Surgery, Baylor College of Medicine, Houston, TX (Y.L., H.G.V., Y.H.S., S.A.L.).; 4 Geisinger Research Institute and Heart & Vascular Institute, Geisinger Medical Center, Danville, PA (S.A.L.).; 5 Université Côte d’Azur, INSERM U1065, France (J.R.).; 6 Division of Cardiovascular Medicine, Victor Phillip Dahdaleh Heart and Lung Research Institute, University of Cambridge, United Kingdom (Z.M.).; 7 Department of Physiology, Saha Cardiovascular Research Center, Saha Aortic Center, University of Kentucky, Lexington (H.S.L., A.D.).

**Keywords:** aortic aneurysm, fibrosis, macrophages, natriuretic peptide, C-type, vascular remodeling

## Abstract

**BACKGROUND:**

Thoracic (TAA) and abdominal (AAA) aortic aneurysm are life-threatening diseases characterized by dilation, inflammation, and structural weakness; development of pharmacological therapies is desperately needed. CNP (C-type natriuretic peptide) plays a key role in vascular homeostasis, mediating vasodilator, anti-inflammatory, and antiatherogenic actions. Since such processes drive AA, we determined the role of endogenous CNP in offsetting pathogenesis.

**METHODS:**

Tissue from patients with AA was analyzed to determine the consequences on CNP signaling. Ascending and suprarenal aortic diameters were assessed at baseline and following Ang II (angiotensin II; 1.44 mg/kg per day) infusion in wild-type, endothelium-restricted (ecCNP^−/−^), fibroblast-restricted (fbCNP^−/−^), global CNP (gbCNP^−/−^), or global NPR-C^−/−^ mice infected with an adeno-associated virus expressing a proprotein convertase subtilisin/kexin type 9 gain-of-function mutation or backcrossed to an apoE^−/−^ background. At 28 days, aortas were harvested for RT-qPCR (quantitative reverse transcription polymerase chain reaction) and histological analyses. CNP (0.2 mg/kg per day) was infused to rescue any adverse phenotype.

**RESULTS:**

Aneurysmal tissue from patients with TAA and AAA revealed that CNP and NPR-C (natriuretic peptide receptor-C) expression were overtly perturbed. ecCNP^−/−^, fbCNP^−/−^, and gbCNP^−/−^ mice exhibited an aggravated phenotype compared to wild-type animals in both ascending and suprarenal aortas, exemplified by greater dilation, fibrosis, elastin degradation, and macrophage infiltration. CNP and NPR-C expression was also dysregulated in murine thoracic AA and abdominal AA, accompanied by increased accumulation of mRNA encoding markers of inflammation, extracellular matrix remodeling/calcification, fibrosis, and apoptosis. CNP also prevented activation of isolated macrophages and vascular smooth muscle cells. An essentially identical phenotype was observed in NPR-C^−/−^ mice and while administration of CNP protected against disease severity in wild-type animals, this phenotypic rescue was not apparent in NPR-C^−/−^ mice.

**CONCLUSIONS:**

Endothelium- and fibroblast-derived CNP, via NPR-C activation, plays important roles in attenuating AA formation by preserving aortic structure and function. Therapeutic strategies aimed at mimicking CNP bioactivity hold potential to reduce the need for surgical intervention.

**Highlights**

- CNP (C-type natriuretic peptide) expression/release is increased in the aortic tissue of patients with aortic aneurysm (AA) and mice with experimental AA as an intrinsic defense mechanism.
- Genetic deletion of CNP from endothelial cells or fibroblasts exacerbates disease severity in murine experimental AA.
- Therapeutic administration of CNP prevents disease progression through activation of cognate NPR-C (natriuretic peptide receptor-C).
- CNP signaling via NPR-C triggers innate protective mechanisms that prevent the development and progression of thoracic and abdominal AA.
- Therapeutic interventions targeting CNP or NPR-C may represent a tangible means to treat AA pharmacologically, preventing dissection and rupture, and reducing the need for surgical intervention.

**See accompanying editorial on page 1064**

Aortic aneurysm (AA) is a multifactorial vascular disorder characterized by ballooning of the aorta that, without treatment, ultimately leads to lethal dissection or rupture. The condition accounts for almost 200 000 deaths per annum worldwide,^1,2^ and this already significant mortality is likely to grow in the future due to the expanding aging population and increased prevalence of cardiometabolic disease. AA can form in any aortic segment, including the ascending thoracic AA (TAA) or abdominal AA (AAA) regions, and is characterized by chronic inflammation, hemodynamic stress, and structural weakness.^3^ It is common in patients with connective tissue disorders (eg, Marfan syndrome [MFS])^4,5^ and is more prevalent in male than in female patients.^3,6^ The pathogenesis of AAA is distinct to TAA,^3^ although they share common risk factors (eg, smoking, hypertension, age, sex, dyslipidemia, and family history), and the defining mechanisms remain unknown.^7^ Treatment for AA relies on surgery (ie, endovascular or open surgery), itself associated with a significant risk of complications and mortality.^1^ Clinical trials using angiotensin-converting enzyme inhibitors, β-blockers, statins, or antibiotics have not shown significant benefit in treating AA,^8^ although angiotensin receptor blockers display modest efficacy in MFS.^9^ A pharmacological intervention for AA is, therefore, a substantial, unmet medical need.

CNP (C-type natriuretic peptide) is a key mediator involved in preserving vascular reactivity and integrity,^10–14^ governing processes including vasodilation, angiogenesis, and remodeling. In accord, genetic disruption of CNP in endothelial cells and pericytes leads to hypertension and atherogenesis.^10,11,13^ Indeed, in mice with endothelium-restricted deletion of CNP, we reported that male mice exhibit spontaneous thoracic and abdominal aortic dilatations.^13^ These key observations provide pathophysiological context to earlier reports describing beneficial pharmacological effects of CNP in neointimal and medial hyperplasia.^15,16^ Moreover, CNP expression is upregulated in human atherosclerotic lesions and in the culprit vessel following percutaneous coronary intervention^17^; such changes are driven, in part, by TGF-β (transforming growth factor-β).^18,19^ Interestingly, NPR-B^−/−^ (natriuretic peptide receptor) mice exhibit aortic valve calcification and ascending aortic dilation,^20^ suggesting that this cognate receptor contributes to the beneficial actions of CNP in maintaining vascular structure; this is corroborated by the antiproliferative, antimigratory function of this NPR in vascular smooth muscle cells (VSMCs).^21^ However, in sharp contrast, NPR-C–triggered mechanisms are responsible for CNP-dependent regulation of microvascular resistance, immune cell reactivity, platelet activation, angiogenesis, and vascular remodeling.^13,22^ NPR-C is also upregulated in atherosclerotic lesions,^23^ and human genomic studies have shown associations between NPR-C and susceptibility to AA risk factors.^24,25^ Collectively, these observations hint that CNP is critical to processes that promote AA formation; however, a paucity of information exists as to a potential role for CNP in the pathogenesis of AA and the underpinning mechanisms, including the critical NPRs responsible.

To address this deficit in understanding, herein, we utilized well-validated murine models of TAA and AAA,^26,27^ unique global^28^ and cell-specific^12–14^ CNP-null mice, and clinical samples, to study the role of CNP in the initiation and progression of AA in both the thoracic and abdominal aorta. We further explored the potential of targeting cognate NPRs pharmacologically to protect against the pathogenic consequences of AA.

## Materials and Methods

All study data are included in the main text and Supplemental Material; all data, analytical methods, and study materials can be made available upon reasonable request. Additional methodological details and a Major Resources Table can be found in the Supplemental Material.

### Human Aortic Samples

Thoracic and abdominal aortic samples were obtained from patients at Baylor College of Medicine, University College London-Royal Free Hospital Biobank, and University Hospital of Nice and stored at −80 °C until RNA extraction. Patient demographic characteristics are detailed in Tables 1 and 2.

**Table 1.**
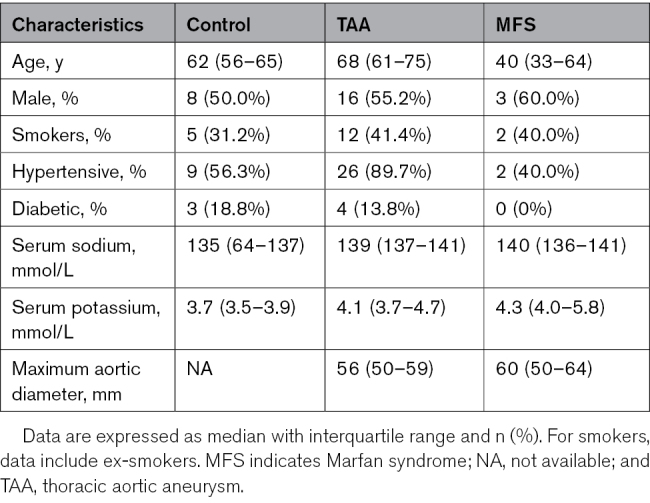
Demographic Data for the Patients With Sporadic Ascending TAA and MFS

**Table 2.**
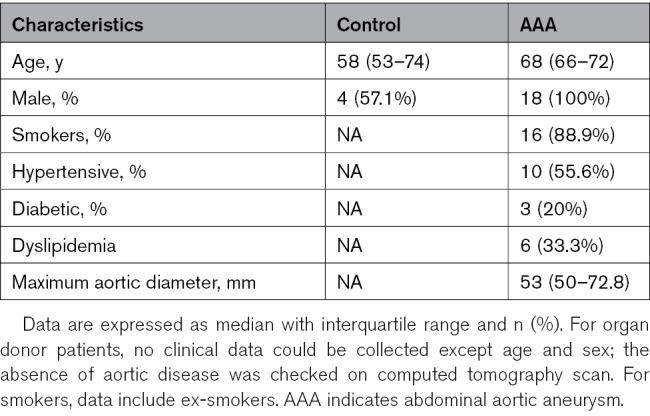
Demographic Data for the Patients With AAA

#### Ascending Aortic Samples

Control ascending aortic tissue samples were obtained from donors or recipients of heart or lung transplants without aortic disease, and diseased aortic tissue samples were obtained from patients with MFS or sporadic ascending TAA.

#### Abdominal Aortic Samples

Infrarenal abdominal aorta was obtained from patients undergoing elective open AAA repair. The diagnosis of AAA is defined as a dilatation of the abdominal aorta with a diameter >50 mm based on computed tomography scans. Healthy abdominal aortic tissue was obtained from organ donors.

### Ang II–Induced Ascending and AAA

Male mice (10–16 weeks of age; C57BL/6J background) were randomly assigned to interventions and the experimenter blinded to treatment or genotype wherever feasible. Due to the lack of AAA formation in female mice infused with Ang II (angiotensin II)^29^ (although females do exhibit thoracic aortic dissection in this model^30^), and the significantly greater prevalence of both TAA and AAA in males in the human population,^6^ only male mice were studied herein.

#### Pretreatment

Before aneurysm induction (Ang II infusion), ecCNP^−/−^, gbCNP^−/−^, fbCNP^−/−^, and NPR-C^−/−^ mice and wild-type (WT) littermates were injected (intraperitoneally) with adeno-associated virus (AAV) vectors containing inserts expressing a mouse PCSK9^D377Y^ gain-of-function mutation (equivalent to human PCSK9^D374Y^ gain-of-function mutation), as described previously.^26^ This is referred to AAV.mPCSK9^D377Y^ in this article. Mice were subsequently fed a Western diet containing saturated fat (TD88137, Teklad Diet; Envigo) for 2 weeks before Ang II implantation.^26^ The Western diet was continued during Ang II infusion. Mice on an ApoE^−/−^ background (ecCNP^−/−^/ApoE^−/−^ and NPR-C^−/−^/ApoE^−/−^) received no pretreatment and had access to normal chow (PicoLab mouse diet 20 EXT; IPS Product Supplies, Ltd).

#### Ang II Infusion

Mice were infused subcutaneously with implanted osmotic mini-pump (1004; Alzet) containing Ang II (1.44 mg/kg per day, H-1705; Bachem) for 28 days as described previously.^26,31^ Briefly, mice were anesthetized (1.5%–2% isoflurane), and body temperature was regulated using a heated mat with a feedback loop (TCAT-2LV controller; Physitemp). An osmotic mini-pump was implanted subcutaneously in the left flank of the mice. Incisions were closed using 6-0 sutures (W-812; Ethicon). Postoperative pain was alleviated by administration of analgesic (buprenorphine, 0.1 mg/kg, Vetergesic; Alstoe Animal Health). In some experiments, mice also received osmotic mini-pump (Alzet Model 1004; Durect Corp) containing CNP (0.2 mg/kg per day)^12–14^ or atrial natriuretic factor-des[Gln18, Ser19, Gly20, Leu21, Gly22] ANF4-23-NH2 (cANF^4–23^) (0.4 mg/kg per day)^28^ for 28 days.

### mRNA Quantification, Immunohistochemistry, and Immunostaining

These were conducted according to standard protocols. Further information can be found in the Supplemental Material.

### Data Analysis

All data are expressed as mean±SEM apart from patient demographic data in Tables 1 and 2, which are expressed as median and interquartile range. Statistical analyses were performed using GraphPad Prism (version 10; GraphPad Software, CA) after confirming normal distribution of each data set using a Shapiro-Wilk test. Two-tailed unpaired Student *t* test, 1-way ANOVA, or 2-way ANOVA (with and without repeated measures, where appropriate) followed by a Sidak multiple comparisons test (with adjustment for multiplicity) was used where appropriate. Statistical significance was defined as *P*<0.05, and the *P* values presented in each Figure indicate all comparisons undertaken.

## Results

### Perturbed Expression of CNP and Cognate NPRs in Human and Murine AAs

**Figure 1.**
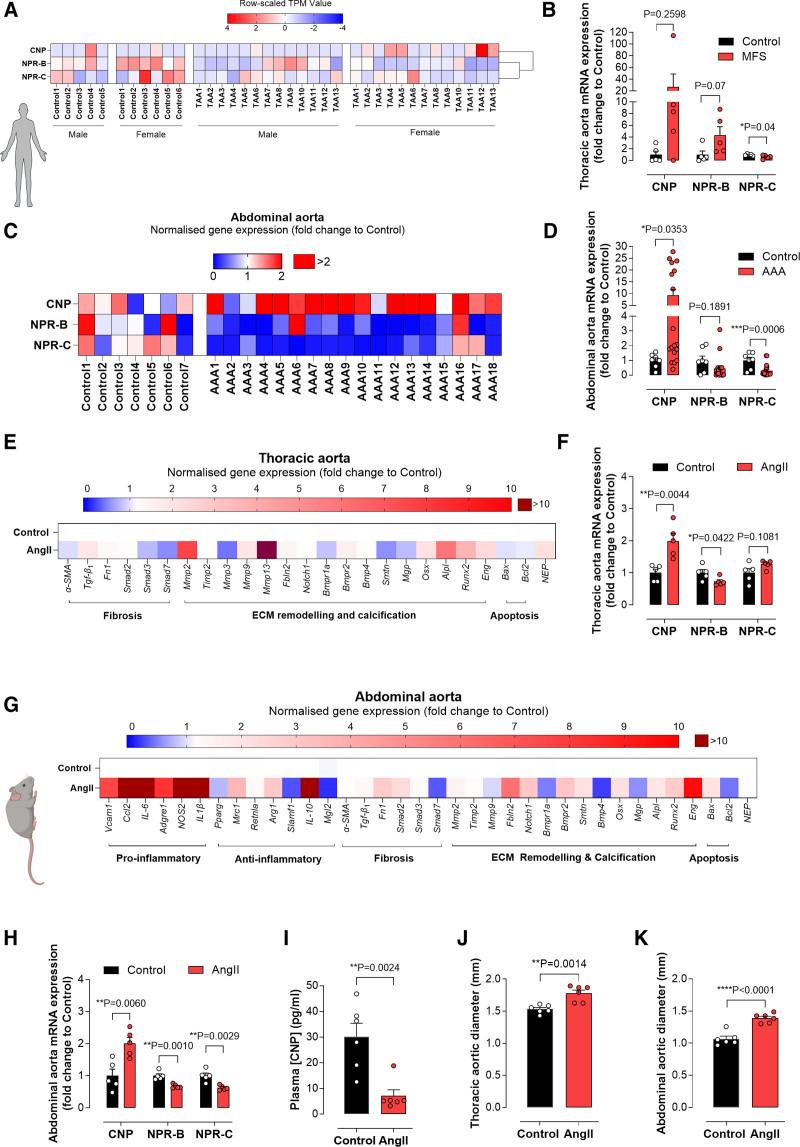
**Perturbed CNP (C-type natriuretic peptide) and NPR (natriuretic peptide receptor) expression in human and murine thoracic aortic aneurysm (TAA) and abdominal aortic aneurysm (AAA).** Individual heatmap showing quantification of RNA-seq data from human aortic media of control and sporadic ascending TAA (**A**), relative mRNA expression of CNP, NPR-B, and NPR-C in aortic samples from patients with nonaortic aneurysm-related disease (control) and individuals with Marfan syndrome (MFS) or AAA (**B**–**D**). Heatmaps (**E** and **G**) and quantified data (**F** and **H**) indicating the relative mRNA expression of markers of fibrosis, inflammation, and apoptosis, and CNP, NPR-B, and NPR-C in aortic samples from ApoE^−/−^ mice in the absence (control) and presence of an Ang II (angiotensin II) infusion (1.44 mg/kg per day; 28 days); plasma CNP concentrations (**I**) and thoracic (**J**) and abdominal (**K**) aortic diameter in the same animals. Data are represented as mean±SEM. Statistical analysis by 1-way ANOVA with Sidak post hoc test (**B**, **D**, **F**, and **H**) or 2-tailed Student *t* test (**I**, **J**, and **K**). Each statistical comparison undertaken has an assigned *P* value (adjusted for multiplicity). n=5 to 18 patients or animals per group.

Analysis of human tissue from sporadic ascending TAA and control subjects revealed that CNP, NPR-B, and NPR-C are expressed in the aortic media (Figure 1A). To corroborate these findings, we analyzed the abundance of total mRNA in ascending aortic biopsies from patients with MFS. Here, ascending aortic NPR-C mRNA abundance was significantly reduced compared with healthy controls (Figure 1B). In contrast, the expression of CNP and NPR-B mRNA was statistically unchanged, although the mean abundance of CNP mRNA was >25-fold greater than controls (Figure 1B). In aneurysmal tissue from patients with AAA, CNP mRNA abundance increased, whereas NPR-C mRNA abundance was reduced (Figure 1C and 1D); NPR-B mRNA abundance did not change significantly (Figure 1B and 1D). These data intimate that an upregulation of CNP expression, but corresponding downregulation of NPR-C expression, might make an important contribution to the pathogenesis of both TAA and AAA (although the changes in CNP expression, especially in TAA, appear to be driven by a subset of patients; it is not clear why this is the case).

In parallel, we used the well-validated Ang II model^26,27^ to uncover the molecular mechanisms underlying the pathogenesis of AA. Ang II infusion (28 days) caused a significant increase in the mRNA abundance of profibrotic (eg, *Tgf-β*_*1*_) and proremodeling/calcification (eg, *Mmp2*, *Mmp13*, *Bmpr2*, *Osx*, *Alpl*, *Runx2*, and *Eng*) pathways in the thoracic aortic region compared with control ApoE^−/−^ mice (Figure 1E; Figure S1A), commensurate with previous work.^19,27,32^ This was accompanied by an increase in CNP mRNA abundance; in this setting, NPR-B mRNA abundance was reduced while NPR-C mRNA remained unchanged compared with controls (Figure 1F). Mirroring these findings and observations in human tissue, Ang II infusion upregulated an analogous panel of genes involved in inflammation, fibrosis, apoptosis, ECM remodeling, and calcification in the abdominal aortic region (Figure 1G; Figure S1B); these data also dovetail well with previous work using this model.^4,27^ Akin to observations in human aneurysmal tissue, in murine AAA, CNP mRNA abundance was increased but NPR-B and NPR-C mRNA was reduced significantly (Figure 1H). Interestingly, in the Ang II model, while CNP mRNA abundance was increased locally in the aneurysmal tissue, circulating CNP concentrations were reduced (Figure 1I). These data indicate that CNP degradation by peptidases might be expedited in the inflammatory environment of AA, commensurate with increased NEP mRNA abundance in diseased aorta, at least in the thoracic region (Figure 1E); certainly, the consistent downregulation of NPR-C mRNA abundance in human and murine AA infers that increased clearance of CNP, a perceived function of NPR-C,^33^ does not appear to underlie the lower plasma peptide concentrations. Finally, the generation of both TAA and AAA in this murine model was confirmed by measurement of the ascending (Figure 1J) and abdominal (Figure 1K) aortic diameter. In sum, these findings reveal relatively consistent changes in CNP and NPR-C mRNA abundance in human and murine TAA and AAA, whereas NPR-B mRNA abundance was inconsistent, perhaps arguing that CNP/NPR-C signaling is critical to host defense in offsetting the pathogenesis of AA. It is also possible that some inconsistency in mRNA abundance was due to the disparity between end-stage disease in patients with AA compared with less severe pathogenesis in Ang II–induced murine models of AA.

### Exacerbated TAA and AAA in gbCNP^−/−^ Mice

**Figure 2.**
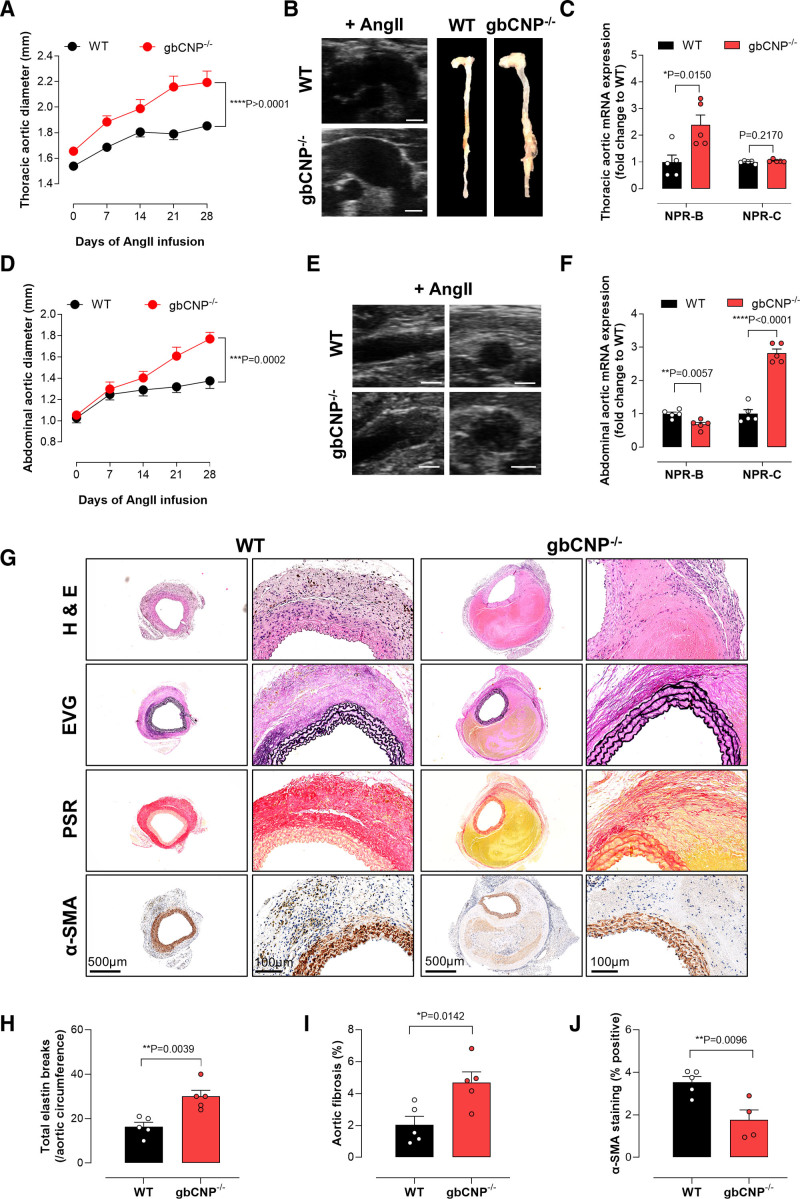
**Global CNP (C-type natriuretic peptide) deletion exacerbates thoracic aortic aneurysm (TAA) and abdominal aortic aneurysm (AAA).** Thoracic (**A**–**C**) and abdominal (**D**–**F**) aortic diameter (**A** and **D**), representative ultrasound and full-length aortic images (**B** and **E**), and relative mRNA expression of NPRs (natriuretic peptide receptors; NPR-B and NPR-C) in aortic samples from wild-type (WT) mice and global CNP-null animals (gbCNP^−/−^) pretreated with AAV.mPCSK9^D377Y^ and fed a Western diet, in the presence of an Ang II (angiotensin II) infusion (1.44 mg/kg per day; 28 days); representative immunohistochemical images of the aortic wall in WT and gbCNP^−/−^ mice following Ang II infusion (**G**) with quantification of elastin degradation (**H**), fibrosis (**I**), and α-SMA (α-smooth muscle actin; **J**; scale bar, 500 or 100 µm). Data are represented as mean±SEM. Statistical analysis by 2-way ANOVA with repeated measures (**A** and **D**), 1-way ANOVA with Sidak post hoc test (**C** and **F**), or 2-tailed Student *t* test (**H**–**J**). Each statistical comparison undertaken has an assigned *P* value (adjusted for multiplicity). n=4 to 10 animals per group. EVG indicates elastic van Gieson; H&E, hematoxylin and eosin; and PSR, picrosirius red.

To investigate a broader role for CNP in AA, we used mice with a global deletion of CNP (gbCNP^−/−^).^28^ Ang II–infused gbCNP^−/−^ mice exhibited a greater increase in ascending thoracic aortic diameter compared with WT (Figure 2A and 2B), with no significant change in blood pressure (Table 3). Gene expression analysis in the thoracic aortic tissues revealed enhanced vascular fibrosis (eg, *Tgf-β*_*1*_, *Fn1*, and smad family member [*Smad2/3/7*]), ECM remodeling (eg, *Mmp2*), calcification (*Bmpr2*, *Osx*, and *Notch1*), and apoptosis (*Bax* and *Bcl2*; Figures S2A and S4). NPR-B mRNA abundance was increased significantly but NPR-C mRNA remained unchanged (Figure 2C).

**Table 3.**
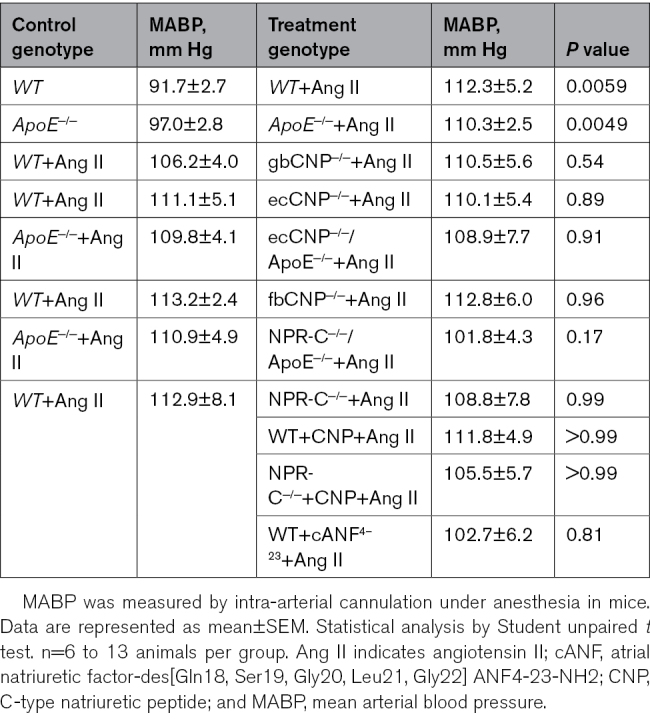
Mean Arterial Blood Pressure Under Anesthesia in the Transgenic Strains Utilized in This Study

Similarly, the progression and development of AAA in the gbCNP^−/−^ mice was accelerated post-Ang II infusion compared with WT mice, indicative of a greater disease severity (Figure 2D and 2E; Figure S3). While NPR-B mRNA was reduced in the abdominal aorta of Ang II–infused gbCNP^−/−^ mice, there was a significant increase in NPR-C mRNA compared with WT (Figure 2F). Ang II infusion caused enhanced medial thickening as the AAA forms in gbCNP^−/−^ mice with luminal expansion and increased collagen deposition (picrosirius red staining; Figure 2G). This was accompanied by increased abundance of genes that promote vascular remodeling, fibrosis, calcification, and apoptosis (Figures S2B and S4). The ECM structure was also disorganized, suggesting a switch of the VSMCs from a contractile to synthetic phenotype (*Mmp-9* and *Smtn*; Figures S2B and S4). The integrity of the abdominal wall was also impaired in gbCNP^−/−^ (Figure 2G), with increased elastin degradation (*Mmp2*, elastic van Gieson staining; Figure 2H), fibrosis (picrosirius red staining; Figure 2I), and reduced α-SMA (α-smooth muscle actin) expression (Figure 2J). Indeed, comparable changes in the profibrotic, proremodeling/calcification and apoptotic gene expression profile were observed in TAA and AAA across all strains with cell-restricted deletion of CNP (compared with WT; Figures S2 and S4). Furthermore, Ang II–induced reductions in plasma CNP and cGMP levels were equivalent between WT mice and animals with cell-restricted CNP deletion (Figure S5A through S5E).

In concert with previous findings, these findings suggest that CNP/NPR-C signaling is critical to the structural and functional integrity of the blood vessel wall.^13,34^ Moreover, these data highlight a multifunctional role of CNP in the pathogenesis of both Ang II–induced TAA and AAA.

### Endothelial-Restricted Deletion of CNP Cells Augments Ang II–Induced TAA and AAA

**Figure 3.**
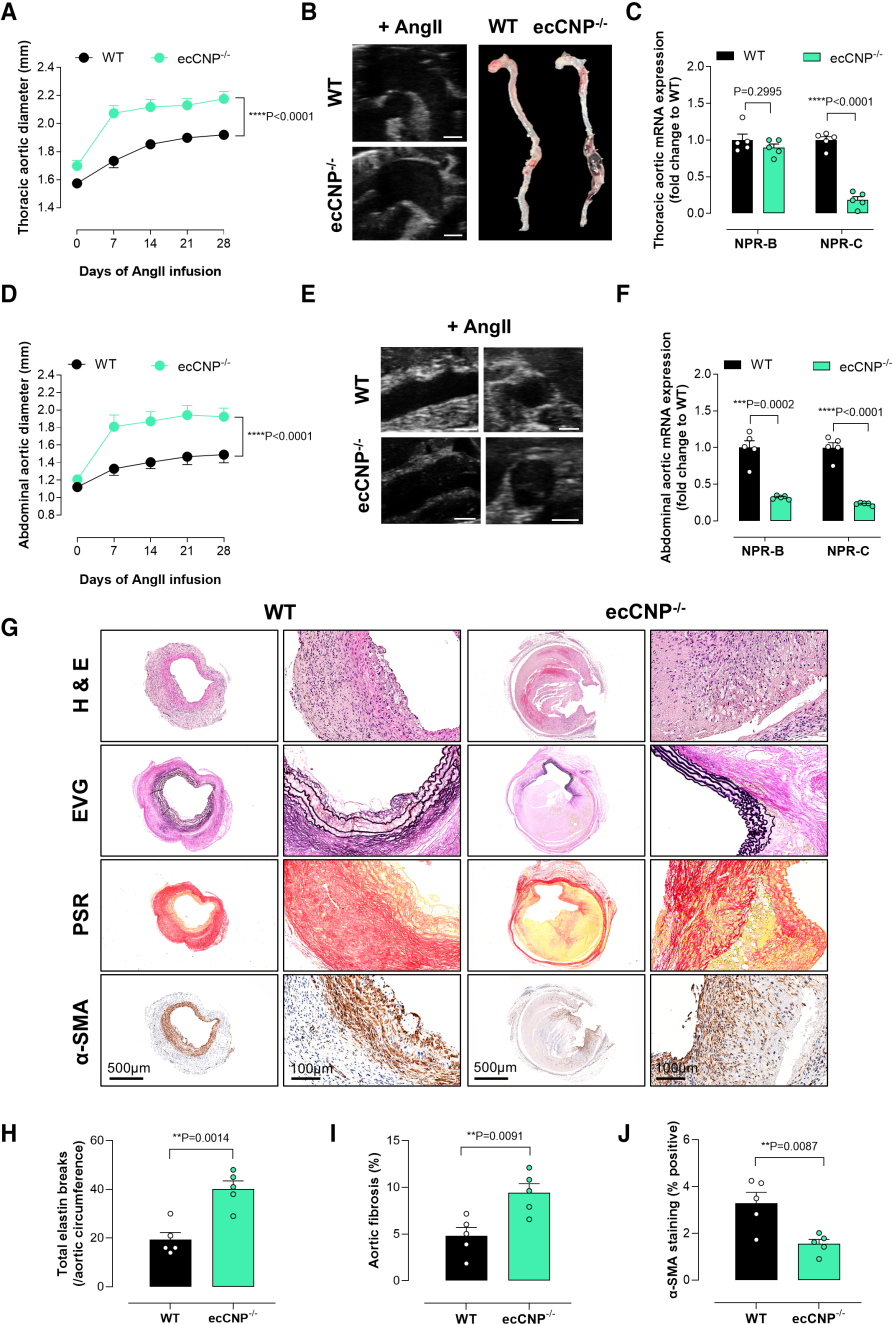
**Endothelium-restricted CNP (C-type natriuretic peptide) deletion exacerbates thoracic aortic aneurysm (TAA) and abdominal aortic aneurysm (AAA).** Thoracic (**A**–**C**) and abdominal (**D**–**F**) aortic diameters (**A** and **D**), representative ultrasound and full-length aortic images (**B** and **E**), and relative mRNA expression of NPRs (natriuretic peptide receptors; NPR-B and NPR-C) in aortic samples from wild-type (WT) mice and endothelium-specific CNP-null animals (ecCNP^−/−^) on an ApoE^−/−^ background in the presence of an Ang II (angiotensin II) infusion (1.44 mg/kg per day; 28 days); representative immunohistochemical images of the aortic wall in WT and ecCNP^−/−^ mice following Ang II infusion (**G**) with quantification of elastin degradation (**H**), fibrosis (**I**), and α-SMA (α-smooth muscle actin; **J**; scale bar, 500 or 100 µm). Data are represented as mean±SEM. Statistical analysis by 2-way ANOVA with repeated measures (**A** and **D**), 1-way ANOVA with Sidak post hoc test (**C** and **F**), or 2-tailed Student *t* test (**H**–**J**). Each statistical comparison undertaken has an assigned *P* value (adjusted for multiplicity). n=5 to 9 animals per group. EVG indicates elastic van Gieson; H&E, hematoxylin and eosin; and PSR, picrosirius red.

To determine which cell types were key to the synthesis and release of CNP in TAA and AAA, we first utilized a mouse strain with endothelium-restricted deletion of CNP^13^ (crossed to an ApoE^−/−^ background) because this is the major cellular source of CNP in the vasculature.^13,18^ Ang II–induced aortic expansion was enhanced in the ascending thoracic aorta of ecCNP^−/−^/ApoE^−/−^, independent of blood pressure (Figure 3A and 3B; Table 3). A similar phenotype was observed in Ang II–infused ecCNP^−/−^ mice administered with AAV.mPCSK9^D377Y^ and fed a Western diet (Figure S5; Table 4). Ang II–induced reductions in plasma CNP and cGMP levels remained unchanged in WT and ecCNP^−/−^ (Figure S5C and S5D). While NPR-B mRNA abundance remained unchanged, NPR-C mRNA was downregulated with enhanced abundance of genes associated with fibrosis (*Tgf-β*_*1*_ and *Fn1*) and ECM remodeling/calcification (*Runx2*) in the ascending aorta of ecCNP^−/−^/ApoE^−/−^ mice (Figure 3C; Figure S2A). There was also significant increased fibulin-2 mRNA abundance (an ECM protein important during tissue repair) in ecCNP^−/−^/ApoE^−/−^ mice, indicative of possible VSMC phenotypic modulation and migration in TAA in mice with endothelium-specific CNP deletion (Figures S2A and S4).

**Table 4.**
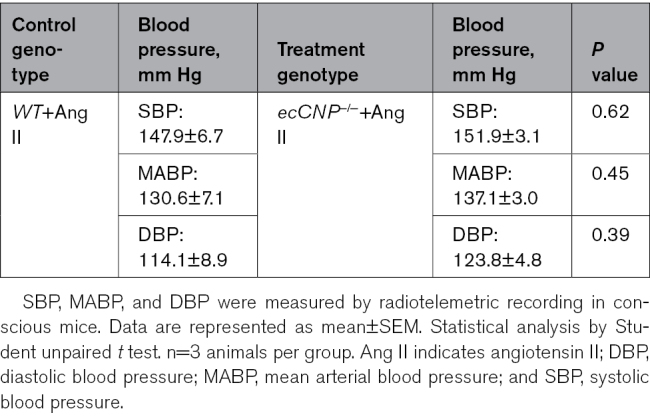
Conscious Blood Pressure Recordings in the Transgenic Strains Utilized in This Study

The development and progression of AAA were also accelerated markedly in ecCNP^−/−^/ApoE^−/−^ mice and ecCNP^−/−^ mice administered AAV.mPCSK9^D377Y^ typified by increased aortic diameter (Figure 3D and 3E; Figures S3 and S5) independent of changes in blood pressure (Tables 3 and 4), supporting our earlier observations that endothelial CNP is protective in AA.^13^ This was accompanied by a significant reduction in both NPR-B and NPR-C mRNA abundance in abdominal aorta of Ang II–infused ecCNP^−/−^/ApoE^−/−^ compared with WT mice (Figure 3F). Similar to the gbCNP^−/−^ mice, ecCNP^−/−^/ApoE^−/−^ mice showed increased gene abundance of mediators that are profibrotic (*Tgf-β*_*1*_ and *Fn1*), proapoptotic (*Bax/Bcl2*), and that promote ECM remodeling/calcification (*Fbln2*, *Notch1*, and *Mmp2*) in the aneurysmal abdominal aortas (Figures S2B and S4). The most abundant genes in the Ang II–infused ecCNP^−/−^/ApoE^−/−^ mice are *Notch1* and *Runx2*, again highlighting an important role of CNP in regulating vessel calcification. Histological analysis showed increased fibrosis in the aneurysmal abdominal aorta, with interstitial collagenous (picrosirius red staining) matrix of the aortic medial layer, increase in elastin degradation, and reduction in α-SMA expression (Figure 3G through 3J; Figure S4). In concert, these findings establish that endothelium-derived CNP protected against vascular pathologies including AA, dampening development of fibrosis, ECM degradation, and calcification.

### Fibroblast-Restricted Deletion of CNP Cells Exacerbates Ang II–Induced TAA and AAA

**Figure 4.**
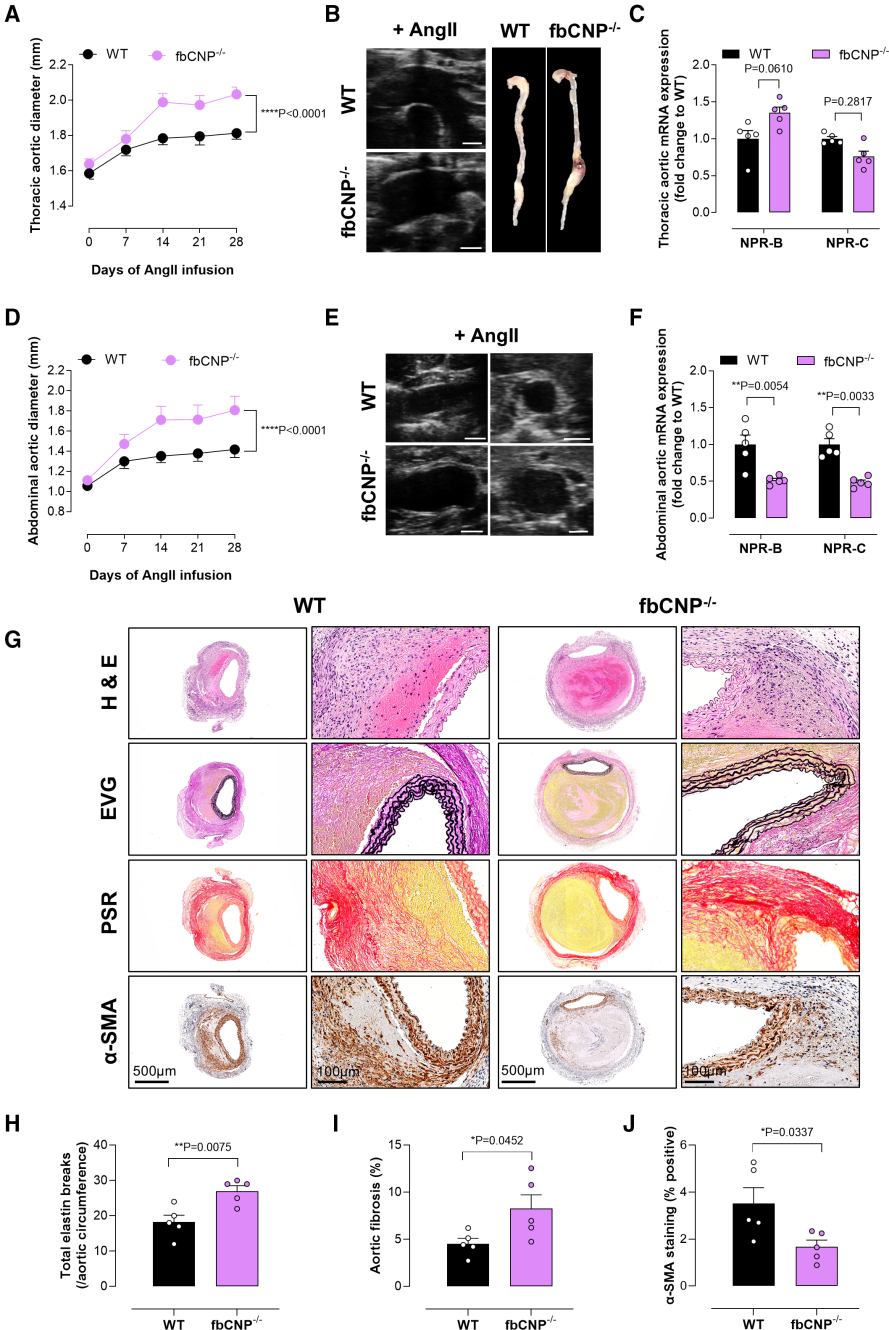
**Fibroblast-restricted CNP (C-type natriuretic peptide) deletion exacerbates thoracic aortic aneurysm (TAA) and abdominal aortic aneurysm (AAA).** Thoracic (**A**–**C**) and abdominal (**D**–**F**) aortic diameters (**A** and **D**), representative ultrasound and full-length aortic images (**B** and **E**), and relative mRNA expression of NPRs (natriuretic peptide receptors; NPR-B and NPR-C) in aortic samples from wild-type (WT) mice and fibroblast-specific CNP-null animals (fbCNP^−/−^) pretreated with AAV.mPCSK9^D377Y^ and fed a Western diet in the presence of an Ang II (angiotensin II) infusion (1.44 mg/kg per day; 28 days); representative immunohistochemical images of the aortic wall in WT and fbCNP^−/−^ mice following Ang II infusion (**G**) with quantification of elastin degradation (**H**), fibrosis (**I**), and α-SMA (α-smooth muscle actin; **J**; scale bar, 500 or 100 µm). Data are represented as mean±SEM. Statistical analysis by 2-way ANOVA with repeated measures (**A** and **D**), 1-way ANOVA with Sidak post hoc test (**C** and **F**), or 2-tailed Student *t* test (**H**–**J**). Each statistical comparison undertaken has an assigned *P* value (adjusted for multiplicity). n=5 to 10 animals per group. EVG indicates elastic van Gieson; H&E, hematoxylin and eosin; and PSR, picrosirius red.

In addition to endothelial cells, fibroblasts represent a critical cellular source and target of CNP^12,35–37^ and are a key cell type in the pathogenesis of AA.^38,39^ Using our established fibroblast-specific CNP-null mouse line (fbCNP^−/−^),^12^ we next determined the role of fibroblast-generated CNP in the development of TAA and AAA. Ang II–infused fbCNP^−/−^ animals exhibited an increased ascending thoracic aortic diameter compared with WT mice (Figure 4A and 4B), in the absence of any significant change in blood pressure (Table 3). Ang II–induced reductions in plasma CNP and cGMP levels were equivalent in WT and fbCNP^−/−^ animals (Figure S5E and S5F). Interestingly, the cognate receptors were also differentially regulated in Ang II–infused fbCNP^−/−^ mice whereby NPR-B mRNA abundance was increased significantly, and NPR-C mRNA reduced, akin to that found in the human disease (Figure 4C). Analysis of gene abundance in the aneurysmal ascending aorta revealed a marked increase in Smad-2 mRNA abundance in fbCNP^−/−^ mice compared with WT, indicative of a possible relation between the CNP and TGF-β_1_ signaling pathway in maintaining the integrity of the aortic wall. Smad-2 mutations are associated with arterial aneurysms and dissections.^40^ Osteogenic markers (eg, *Osx* and *Alpl*) were also upregulated, highlighting a worsened calcified aneurysmal ascending aorta in the fbCNP^−/−^ mice (Figures S2A and S4).

Similarly, AAA was exacerbated in the fbCNP^−/−^ mice compared with WT mice (Figure 4D and 4E; Figure S3), without any change in blood pressure (Table 3). Expression of the cognate receptors mirrored that in the ascending aorta of fbCNP^−/−^ mice, highlighting the importance of both NPR-B and NPR-C in mediating CNP effects related to fibroblasts (Figure 4F). Similarly, there was an increase in fibrosis (*Tgf-β*_*1*_) and calcification (*Alp* and *Runx2*) in the aneurysmal abdominal aorta of fbCNP^−/−^ mice (Figures S2B and S4). Enhanced expression of MMPs (matrix metalloproteinases) 2 and 9 was associated with degradation of elastin and interstitial collagenous matrix of the medial layer of the aorta (Figure 4G through 4J). The most abundant genes in fbCNP^−/−^ mice were related to ECM remodeling, comprising *Mmp2/9*, *Fbln2*, *Notch1*, and *Bmpr1a* (Figures S2B and S4). Together, these data suggest that paracrine CNP signaling in aortic fibroblasts has antifibrotic and antiremodeling effects in Ang II–induced AA in both ascending and abdominal aortas.

### Global NPR-C Deletion Augments Ang II–Induced TAA and AAA

**Figure 5.**
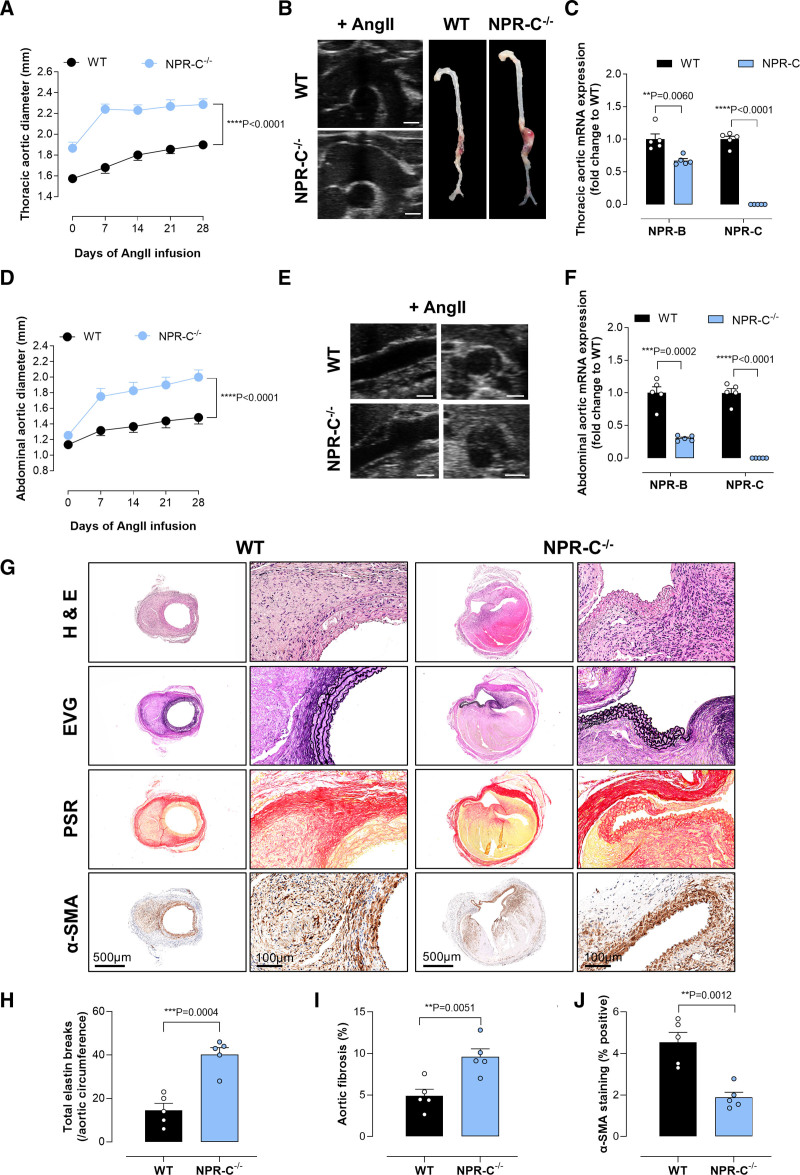
**Global natriuretic peptide receptor (NPR)-C deletion exacerbates thoracic aortic aneurysm (TAA) and abdominal aortic aneurysm (AAA).** Thoracic (**A**–**C**) and abdominal (**D**–**F**) aortic diameters (**A** and **D**), representative ultrasound and full-length aortic images (**B** and **E**), and relative mRNA expression of NPR-B and NPR-C in aortic samples from wild-type (WT) mice and global NPR-C–null animals (NPR-C^−/−^) on an ApoE^−/−^ background in the presence of an Ang II (angiotensin II) infusion (1.44 mg/kg per day; 28 days); representative immunohistochemical images of the aortic wall in WT and NPR-C^−/−^ mice following Ang II infusion (**G**) with quantification of elastin degradation (**H**), fibrosis (**I**), and α-SMA (α-smooth muscle actin; **J**; scale bar, 500 or 100 µm). Data are represented as mean±SEM. Statistical analysis by 2-way ANOVA with repeated measures (**A** and **D**), 1-way ANOVA with Sidak post hoc test (**C** and **F**), or 2-tailed Student *t* test (**H**–**J**). Each statistical comparison undertaken has an assigned *P* value (adjusted for multiplicity). n=5 to 12 animals per group. EVG indicates elastic van Gieson; H&E, hematoxylin and eosin; and PSR, picrosirius red.

To establish the key cognate NPRs that underpinned the protective actions of CNP to offset TAA and AAA development, we utilized mice with a global NPR-C deletion.^12–14,28,33^ Interestingly, NPR-C^−/−^ mice have an elongated stature and long digits at baseline, a phenotype that mirrors MFS patients, hinting at a pathogenic connection. Indeed, NPR-C^−/−^/ApoE^−/−^ mice have a dilated ascending aorta at baseline (Figure 5A), mirroring the phenotype common to individuals with MFS. Ang II infusion augmented ascending thoracic aortic dilation in NPR-C^−/−^/ApoE^−/−^ compared with ApoE^−/−^ (control) mice, without any significant change in blood pressure (Figure 5A and 5B; Table 3). These data support the thesis that NPR-C activation (by CNP) maintains a critical influence on the structural integrity of the ascending aorta. Gene abundance analysis revealed a downregulation in both receptor subtypes in the ascending thoracic aorta of NPR-C^−/−^/ApoE^−/−^ mice compared with controls (Figure 5C). Similar to the findings in gbCNP^−/−^ mice, there was an increase in abundance of genes associated with fibrosis (*Fn1* and *Smad3*), ECM remodeling (*Mmp3/9/13*), and calcification (*Flbn2* and *Runx2*), as well as apoptosis (*Bax* and *Bcl2*; Figures S2A and S4).

Although there was no significant change in basal diameter of the abdominal aorta between NPR-C^−/−^/ApoE^−/−^ and ApoE^−/−^ (control) mice, Ang II infusion accelerated the development and progression of AAA, with a similar profile in gene expression to the ascending aorta (Figure 5D and 5E; Figure S3). The most abundantly expressed genes in the aneurysmal aorta of NPR-C^−/−^/ApoE^−/−^ mice were related to ECM remodeling/calcification (*Mmp2/9*, *Fbln2*, *Bmpr2*, *Bmp4*, *Mgp1*, *Runx2*, and *Eng*) and apoptosis (*Bax* and *Bcl2*), although for the latter, expression of both *Bax* (proapoptotic) and *Bcl2* (antiapoptotic) was increased making interpretation of a functional consequence difficult (Figures S2B and S4). A similar phenotype in terms of gene expression was observed in Ang II–infused NPR-C^−/−^ mice administered AAV.mPCSK9^D377Y^ and fed a Western diet (Figure S7A and S7B), confirming these distinct experimental models drive a similar disease phenotype. Histological examination of stained sections of the aorta revealed clear differences in the medial and adventitial layers of Ang II–infused NPR-C^−/−^/ApoE^−/−^ compared with control mice, with thrombus formation, adventitial remodeling, inflammatory cell infiltration (hematoxylin and eosin), and elastin fragmentation (elastic van Gieson) with marked fibrosis (picrosirius red; Figure 5G through 5I). These findings suggest that NPR-C is essential for preventing progressive growth of AAA and associated pathogenesis and for underpinning the protective functions of CNP.

### Administration of CNP Limits the Progression of TAA and AAA in Response to Ang II via NPR-C Activation

**Figure 6.**
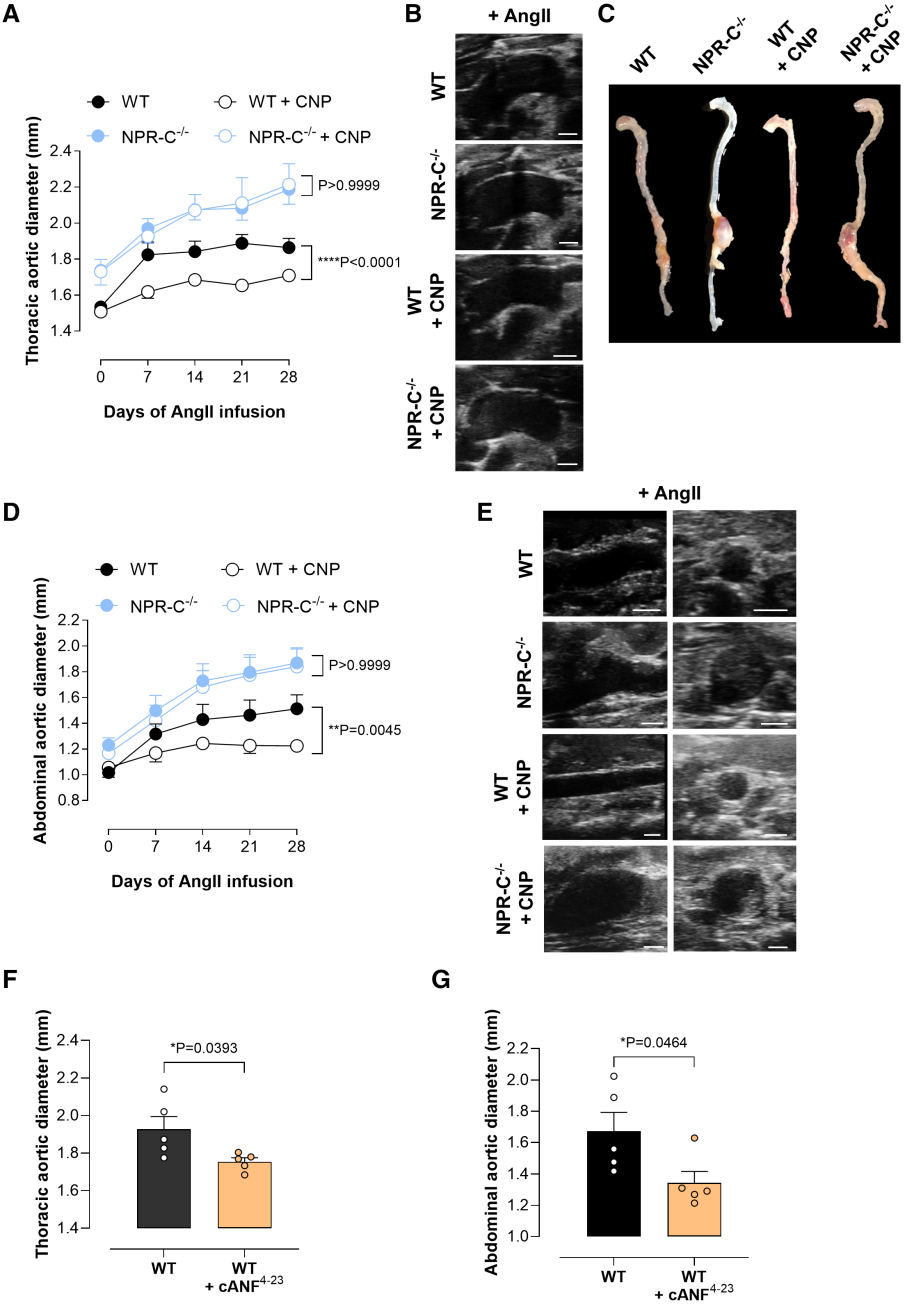
**Therapeutic administration of CNP (C-type natriuretic peptide) or NPR-C (natriuretic peptide receptor-C) agonist prevents disease progression of thoracic aortic aneurysm (TAA) and abdominal aortic aneurysm (AAA) in wild-type (WT) but not in NPR-C^−/−^ mice.** Thoracic (**A**–**C**) and abdominal (**C**–**E**) aortic diameters (**A** and **D**), representative ultrasound (**B** and **E**), and full-length aortic images (**C**) in wild-type (WT) and global NPR-C–null animals (NPR-C^−/−^) pretreated with AAV.mPCSK9^D377Y^ and fed a Western diet in the absence and presence of CNP (0.2 mg/kg per day) or the NPR-C agonist cANF^4–23^ (0.4 mg/kg per day) during concomitant Ang II (angiotensin II) infusion (1.44 mg/kg per day; 28 days). Data are represented as mean±SEM. Statistical analysis by 2-way ANOVA with repeated measures (**A** and **D**) or 2-tailed Student *t* test (**F** and **G**). Each statistical comparison undertaken has an assigned *P* value (adjusted for multiplicity). n=5 to 8 animals per group. cANF indicates atrial natriuretic factor-des[Gln18, Ser19, Gly20, Leu21, Gly22] ANF4-23-NH2.

To confirm that CNP/NPR-C signaling is critical as a host defense mechanism to prevent TAA and AAA and to offer promise that targeting this pathway might be of therapeutic benefit, we examined the effect of CNP administration on progression of AA in Ang II–infused WT and NPR-C^−/−^ mice. Infusion of CNP for 28 days significantly reduced Ang II–induced ascending thoracic and abdominal aortic expansion in WT but not NPR-C^−/−^ animals (Figure 6A through 6E) without a significant change in blood pressure (Table 3). Ang II induced a comparable reduction in plasma CNP and plasma cGMP in WT and NPR-C^−/−^ animals, which was reversed back to WT levels following infusion of CNP (Figure S8A and S8B). These results verify the observations above that CNP-triggered NPR-C activation underlies the salutary actions of the peptide in the setting of AA, as we and others have identified previously in additional cardiovascular disorders.^12–14,41,42^

To further establish the importance of NPR-C in the beneficial actions of endogenous and exogenous CNP in the context of AA, we infused the selective NPR-C peptide agonist cANF^4–23^ for 28 days, which was able to significantly reduce the expansion of the ascending and abdominal aortas of Ang II–infused WT mice (Figure 6F and 6G). Indeed, there was downregulated abundance of several genes related to fibrosis (*Tgf-β*_*1*_ and *Smad2*), ECM remodeling, and calcification (*Mmp2/9*, *Bmpr2*, *Runx2*, and *Eng*), as well as apoptosis (*Bax*) following cANF^4–23^ administration (Figure S2B). Taken together, these findings suggest that CNP administration, or NPR-C activation, at an early stage of the disease reduced the progressive increase in aortic diameter that occurred in AA. These findings suggest that NPR-C activation is able protect against the progression of TAA and AAA by modulating signaling pathways related to fibrosis, ECM remodeling, and apoptosis.

### Protective Effects of CNP/NPR-C Signaling Are Focused on VSMCs and Macrophages

**Figure 7.**
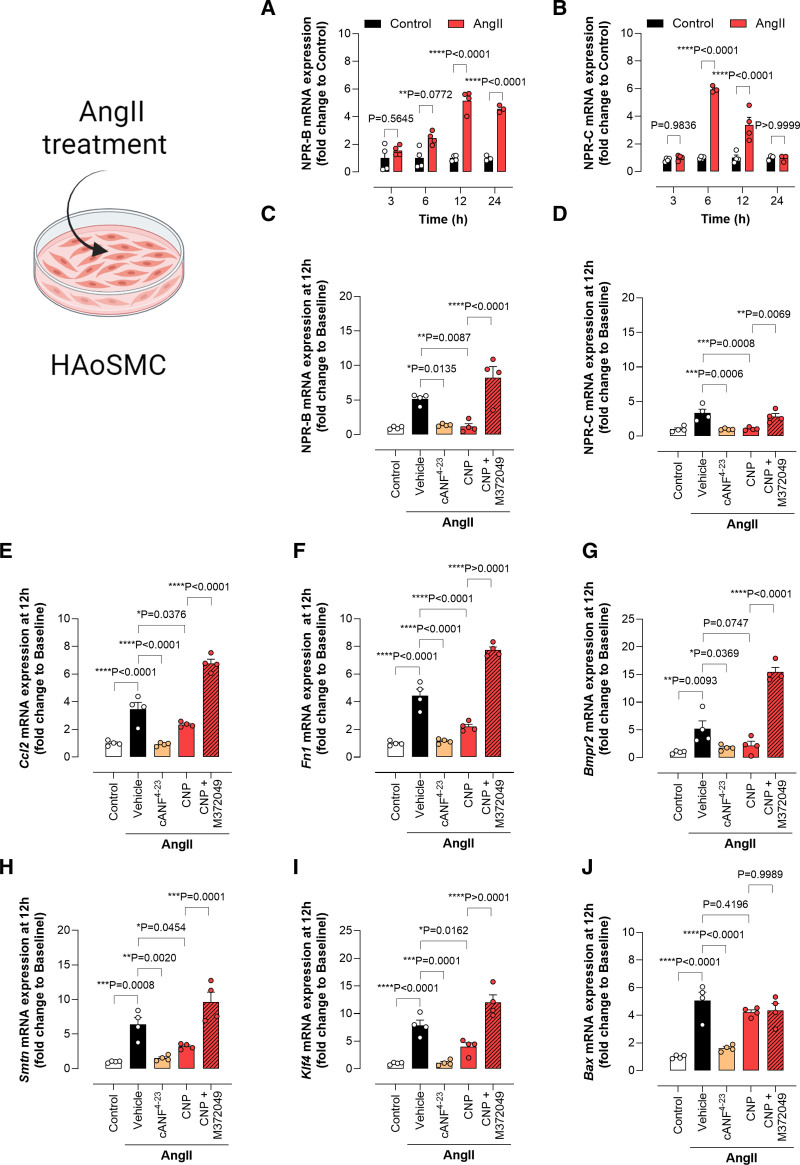
**Markers of inflammation, remodeling, differentiation, and apoptosis are exacerbated by Ang II (angiotensin II) in human aortic smooth muscle cells but rescued by CNP (C-type natriuretic peptide).** Expression of NPRs (natriuretic peptide receptors; NPR-B and NPR-C; **A**–**D**) and markers of inflammation (CCL2 [chemokine C-C motif ligand 2]; **E**), remodeling (fibronectin, **F**; BMPR2 [bone morphogenetic receptor type 2], **G**), phenotypic switching (smoothelin, **H**; Klf4 [Kruppel like factor-4], **I**), and apoptosis (Bax [bcl-2-like protein 4], **J**) in primary human aortic smooth muscle cells in the absence (baseline) and presence of Ang II (200 nmol/L; 3–12 hours, **A** and **B** or at 12 hours, **C**–**J**) with concomitant administration of CNP (100 nmol/L), the NPR-C agonist cANF^4–23^ (200 nmol/L), or CNP plus the NPR-C antagonist M372049 (10 μmol/L). Data are represented as mean±SEM. Statistical analysis by 1-way ANOVA with Sidak post hoc test. Each statistical comparison undertaken has an assigned *P* value (adjusted for multiplicity). n=4 independent experiments per group. cANF indicates atrial natriuretic factor-des[Gln18, Ser19, Gly20, Leu21, Gly22] ANF4-23-NH2.

To elucidate the molecular underpinnings of the effects of CNP on the blood vessel wall, we used healthy human aortic smooth muscle cells (HAoSMC) in culture to determine the signaling pathways responsible for detrimental changes in VSMC structure and function that are key to the development of AA. Ang II administration caused a time-dependent increase in the CNP cognate receptors mRNA abundance, with a peak increase at 6 to 12 hours (Figure 7A and 7B). At 12 hours of Ang II exposure, there was a marked increase in genes associated with various signaling pathways including inflammation (*Ccl2*), fibrosis (*Fn1*), calcification (*Runx2*, *Bmpr2*, and *Smtn*), apoptosis (*Bax*), and SMC phenotyping switching (*Klf4*; Figure 7E through 7J) mimicking the pathogenesis of AA. Interestingly, treatment with CNP or cANF^4–23^ was able to significantly reduce these Ang II–driven effects (Figure 7C through 7J) but not in the presence of the selective NPR-C antagonist M372049.^13^ These results highlight a role of NPR-C in modulating these AA-pathogenic signaling pathways in VSMCs.

**Figure 8.**
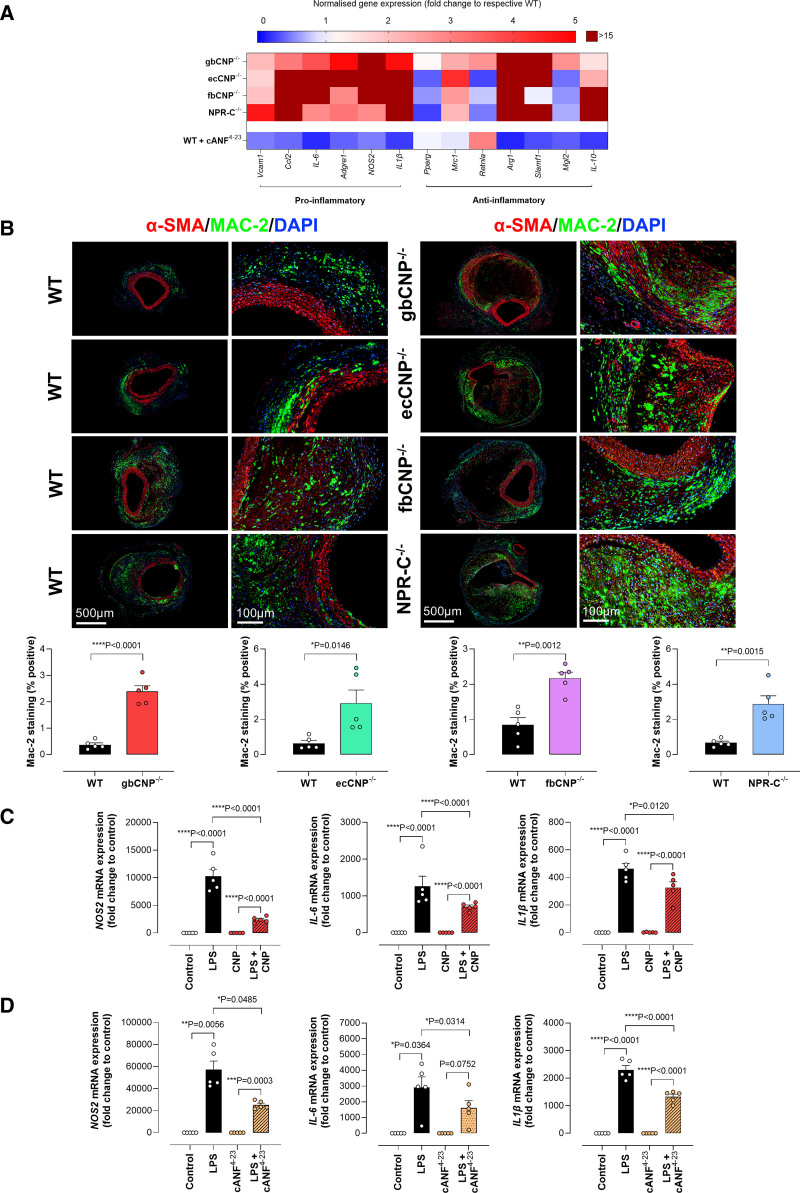
**Genetic deletion of CNP (C-type natriuretic peptide)/NPR-C (natriuretic peptide receptor-C) signaling exacerbates the proinflammatory phenotype in experimental aortic aneurysm (AA) and in isolated macrophages.** Heatmap of markers of inflammation (**A**) and representative immunofluorescence images of macrophage infiltration using anti-Mac-2 (galectin-3) with quantified data (**B**) in wild-type (WT), global (gbCNP^−/−^), endothelium-restricted (ecCNP^−/−^), and fibroblast-restricted (fbCNP^−/−^) CNP-null mice and NPR-C^−/−^ animals following Ang II (angiotensin II) infusion (1.44 mg/kg per day; 28 days; scale bar, 500 or 100 µm). Expression of markers of inflammation (*NOS2*, *IL-6*, and *IL-1*) in isolated murine macrophages in culture in the absence (control) and presence of lipopolysaccharide (LPS; 100 ng/mL) with concomitant administration of CNP (100 nmol/L) or the NPR-C agonist cANF^4–23^ (200 nmol/L; **C** and **D**). Data are represented as mean±SEM. Statistical analysis by 2-tailed Student *t* test (**B**) or 1-way ANOVA with Sidak post hoc test (**C** and **D**). Each statistical comparison undertaken has an assigned *P* value (adjusted for multiplicity). n=5 independent experiments per group. cANF indicates atrial natriuretic factor-des[Gln18, Ser19, Gly20, Leu21, Gly22] ANF4-23-NH2; and DAPI, 4′,6-diamidino-2-phenylindole.

Macrophage infiltration in response to Ang II is crucial in AAA formation and progression in Ang II mouse model of AA.^4^ Global or cell-specific deletion of CNP in both the endothelial cells and fibroblasts increased proinflammatory (*Vcam1*, *Ccl2*, *IL-6*, *Adgre1*, *NOS2*, and *IL1β*) and anti-inflammatory (*Arg1*, *Slamf1*, *Mrc1*, and *IL-10*) markers in the aneurysmal abdominal aortas; global deletion of NPR-C^−/−^ also showed a similar pattern of abundance (Figure 8A). Immunofluorescence staining showed a robust increase in Mac-2 (galectin-3)–positive macrophages in the adventitial and thrombotic area of the aneurysmal abdominal aorta of gbCNP^−/−^, ecCNP^−/−^/ApoE^−/−^, fbCNP^−/−^, and NPR-C^−/−^/ApoE^−/−^ mice as compared with aneurysmal control (Figure 8A and 8B). Interestingly, the anti-inflammatory markers (*PPARγ*, *Retnla*, and *Mgl2*) were upregulated in the abdominal aorta of gbCNP^−/−^ but downregulated in ecCNP^−/−^, fbCNP^−/−^, and NPR-C^−/−^ mice (Figure 8A; Figure S9). These data suggest that the loss of CNP is associated with increased inflammatory responses in AAA. Indeed, administration of cANF^4–23^ reversed this inflammatory phenotype in aneurysmal abdominal aortas of Ang II–infused WT mice (Figure 8A; Figure S9). These data highlight that NPR-C activation can reduce inflammatory markers and elevate anti-inflammatory markers such as *Retnla*^43^ to reduce AAA lesions in Ang II–infused mice.

To further identify the potential pathways by which CNP induces its anti-inflammatory effects, we performed gene expression studies in primary bone marrow–derived macrophages. Lipopolysaccharide/IFN-γ (interferon-γ) increased mRNA abundance of several markers involved in M1 polarization (*NOS2*, *IL-6*, *IL1β*, *Ccl2*, and *Adgre1*) of bone marrow–derived macrophages from WT mice (Figure 8C and 8D). Administration of CNP or cANF^4–23^ significantly reduced mRNA abundance of the inflammatory markers (*NOS2*, *IL-6*, and *IL1β*; Figure 8C and 8D). These data suggest that CNP or NPR-C activation can induce direct anti-inflammatory effects on macrophages to reduce inflammation.

## Discussion

AA is a chronic, life-threatening condition with no effective pharmacological therapy; at present, surgical repair offers the only effective intervention. The disorder is characterized by localized expansion and weakening of the aorta, which is accompanied by adverse remodeling, extracellular matrix decomposition, elastin degradation, immune cell activation and infiltration, phenotypic switching of VSMC, and neovascularization.^3,4,27^ Thus, new pharmacological therapies for AA, which are desperately needed, will ideally target multiple steps within these pathogenic mechanisms that contribute to loss of aortic structural integrity. One endogenous mediator that represents an attractive candidate with respect to offsetting multiple aspects of disease progression in AA is CNP. This paracrine member of the natriuretic peptide family is produced by multiple cell types in the blood vessel wall (eg, endothelial cells and fibroblasts) and exerts a cytoprotective sphere of action that governs vascular reactivity and compliance, medial proliferation and migration, platelet and leukocyte activation, fibrosis, and angiogenesis.^10–14,22,37^ As such, CNP holds potential as both a key intrinsic mediator that slows development and progression of AA and as an effective therapeutic target for this disorder. Herein, we provide compelling evidence to support both these theses and define the underpinning pathways.

Analysis of tissues from patients with both TAA and AAA indicates that there are significant changes in the expression of CNP and its cognate receptors, NPR-B and NPR-C. While NPR-B expression was not affected consistently, there was a common upregulation of CNP and concomitant downregulation of NPR-C mRNA abundance in the aortic tissue from patients with TAA and AAA. This observation infers that the blood vessel wall increases CNP release to counteract the pathogenic processes that drive AA but that one of the key signaling receptors triggered by the peptide, NPR-C is diminished, perhaps negating the salutary actions of CNP. Furthermore, plasma CNP concentrations are reduced in AA despite the increased peptide mRNA abundance, indicating that concentrations of bioactive CNP might be significantly diminished, perhaps by upregulation of peptidases including NEP. However, the localized nature of the AAA lesion suggests that there is not a more generalized CNP deficiency but a focal reduction in bioactivity. An essentially identical phenotype was observed in 2 etiologically distinct murine models of AA,^26,27^ indicating that these experimental systems are good representations of the human disease and that CNP/NPR-C signaling is a shared cross-species cytoprotective pathway. This is consistent with previous work that noted spontaneous AA in male ecCNP^−/−^ mice^13^ and NPR-C mRNA abundance is reduced in human aorta in response to AA risk factors (eg, hypertension and atherosclerosis)^23–25^ and by the similarity in morphological phenotype between global NPR-C^−/−^ mice and patients with MFS.^33,44^ This also mirrors findings that NPR-C activation alters VSMC and migration, extravasation of immune cells, angiogenesis, and remodeling after injury.^14,22,45^ However, recent work has also highlighted a key role for NPR-B in maintaining aortic valve/ascending aortic structural integrity and in the development of atherosclerotic lesions in this region (albeit predominantly in females and not the abdominal area).^20,37^ Interestingly, our data suggest that NPR-B is upregulated in the ascending aorta of patients with MFS but downregulated in the abdominal region of individuals with AAA. This differential expressional change might, therefore, support a more prominent role for NPR-B in the ascending thoracic aorta.

Initial observations confirmed that global deletion of CNP from multiple cell types accelerated the development of AA, compared with WT, in both the ascending thoracic and abdominal aorta in Ang II–infused murine models of AA. The significant increase in aortic diameter over the time course of the models^26,27^ was accompanied by upregulation of various genes that promote fibrosis, ECM remodeling, calcification, and apoptosis. There was also an increased incidence of elastin degradation, greater fibrotic burden, phenotypic switching of VSMC (ie, α-SMA–expressing myofibroblasts), and greater severity of AAA with formation of a false lumen.^27^ This exacerbation of multiple hallmarks of aneurysmal disease confirms the capacity of CNP to dampen many pathways that promote AA pathogenesis. To define the key cell populations that synthesize and release CNP to offset development of AA, we utilized 2 cell-restricted CNP mutant mouse lines: endothelium- (ecCNP^−/−^)^12–14^ and fibroblast (fbCNP^−/−^)-specific deletion.^12^ Indeed, aortic diameter and inflammatory cell accumulation, elastin degradation, fibrosis, calcification, and VSMC phenotypic switching were all exacerbated in both these strains. Such findings intimate that both endothelium- and fibroblast- derived CNP are critical for offsetting the development of AA and each source of CNP mediates multiple, shared beneficial effects on the blood vessel wall to maintain integrity. Indeed, the magnitude of the aggravated disease phenotype in each cell-restricted null mutant was closely matched and similar to the phenotype in mice with global gene deletion. This comparable benefit implies that CNP from both cellular sources is required to exert the optimal salutary activity, perhaps the result of a need to influence various localized pathogenic mechanisms. This concept is corroborated by the finding that global deletion of CNP did not result in an additive/greater disease severity (although the broad upregulation of drivers of pathogenesis observed in gbCNP^−/−^ mice was more extensive than that found in either ecCNP^−/−^ or fbCNP^−/−^ mice). In concert, these data confirm a critical role for CNP, of endothelium and fibroblast origin, in preventing the development of TAA and AAA. Indeed, the fact that CNP deletion aggravated disease severity in both ascending and abdominal regions suggests that the common pathways governed by the peptide are at play in both TAA and AAA, despite the well-validated differences in etiology.^5,46^ This also bodes well for therapeutically targeting CNP-driven pathways in subpopulations of patients with AA since it hints that patient stratification might not be necessary. However, a limitation of this study is that we have only investigated AA formation in male mice (although both sexes were included in the human tissue analysis) and, therefore, we have not established an intrinsic, beneficial role for CNP signaling in females; this clearly warrants further investigation. Indeed, this is particularly pertinent when considering CNP biology because in mice with endothelium-specific deletion of CNP, blood pressure is significantly raised in female but not male animals^13^ (a similar phenomenon has been reported for myocardial fibrosis following pressure overload in fibroblast-restricted NPR-B–deficient animals^37^). This sex difference is underpinned, in part, by greater reliance of females on CNP bioactivity to control vascular resistance and local blood flow (as an EDHF [endothelium-derived hyperpolarizing factor]), whereas males depend more on endothelial NO production.^47^ Whether such a sex difference exists in the human population remains to be determined, but if females use CNP in an analogous manner, this might contribute to the male-oriented prevalence of AA (ie, males are less capable of implementing the CNP-driven protective mechanisms we have identified herein). Such sex-dependent differences are also important in light of the fact that female patients often experience a more rapid disease progression and worse outcome,^6^ so a greater understanding of a potential role for CNP in pathogenic progression in females is undoubtedly needed.

To support our hypothesis that NPR-C, rather than NPR-B, is the prominent cognate receptor triggered by CNP to bring about the salutary actions established above, we explored TAA and AAA formation in global NPR-C^−/−^ mice. In this setting, animals exhibited a baseline ascending aortic expansion that mimics the TAA observed in patients with MFS, which as alluded to previously, is commensurate with the phenotypic similarities between NPR-C^−/−^ mice and MFS.^33^ In addition, NPR-C^−/−^ mice also exhibited a worsened phenotype, with greater severity in AAA characterized by immune cell infiltrate, elastin strand breaks, fibrosis, calcification, and myofibroblast differentiation. The magnitude of these deficits was comparable to that observed in mice with global or cell-restricted deletion of CNP. The gene expression profile in NPR-C^−/−^ mice also revealed overt increase in markers of ECM remodeling, remodeling, and fibrosis, albeit the extent of gene changes was not as broad as that observed in gbCNP^−/−^, ecCNP^−/−^, or fbCNP^−/−^ animals, suggesting that a more focused targeting of CNP activity underpins the beneficial effects of the peptide; specifically, these pathways focus on fibronectin, Smad, MMP, BMP (bone morphogenetic protein), and Runx2 (Runx family transcription factor 2) signaling. These findings clearly implicate NPR-C in the positive action of CNP in offsetting the pathogenesis of AA. Indeed, a predominance of NPR-C versus NPR-B in this regard fits well with previous work in rodent models and patients with AA, highlighting that activation of cGMP-dependent protein kinase (triggered by NPR-B signaling) augments disease progression.^48^

To further confirm the importance of NPR-C to the positive functions of CNP in preventing AA development and to establish whether pharmacologically targeting this NPR holds promise for treatment of the disorder, we explored the ability of CNP and the selective NPR-C agonist cANF^4–23^ to prevent multiple aspects of pathogenesis. Here, CNP reduced aortic expansion in both the ascending and abdominal regions in WT, but importantly not NPR-C^−/−^ mice. In accord, selective NPR-C activation with cANF^4–23^ reduced the phenotype in WT animals. Mirroring these positive structural changes, expression of many of the drivers of pathogenesis that were worsened by CNP gene deletion (as described above) were improved by cANF^4–23^ infusion. These data highlight that activation of NPR-C, either by endogenous CNP or by exogenous peptide agonists, plays a critical role in the beneficial actions of endothelium- and fibroblast-derived CNP in offsetting the pathogenesis of AA and is an effective means by which to pharmacologically recapitulate these beneficial actions of endogenous peptide to treat the disorder. Importantly, even though our data show that NPR-C mRNA abundance is significantly downregulated in murine and human AA, there is still sufficient active receptor present to underpin a pharmacological benefit of NPR-C agonism.

To more fully elucidate the molecular underpinnings of the beneficial activity of CNP/NPR-C signaling in the setting of AA, we focused on 2 key cell types that contribute to AA pathogenesis: VSMCs and macrophages.^4,27,32^ Study of HAoSMC treated with Ang II to mimic the in vivo disease model produces a well-characterized increase in genes that promote remodeling/fibrosis (eg, *Fn1* and *Bmpr2*), inflammation (eg, *Ccl2*), and phenotypic switching (*Smtn* and *Klf4*). Interestingly, this was paralleled by the upregulation of NPR-B and NPR-C mRNA abundance in HAoSMC. Moreover, incubation of HAoSMC with CNP or cANF^4–23^ inhibited the expression of these key drivers of pathogenesis, an effect that could be reversed by the selective NPR-C antagonist M3272049. Such findings establish that CNP triggers NPR-C activation on HAoSMC to directly dampen the inflammatory, proremodeling, and prodifferentiation phenotype triggered by Ang II. This concept dovetails well with previous work, demonstrating an antiatherogenic, antiremodeling effect of CNP.^13–16^ A similar scenario was observed in isolated murine macrophages. Here, activation with lipopolysaccharide elicited the upregulation of genes well-established to characterize a proinflammatory (classically M1) phenotype, including *NOS2*, *IL1β*, and *IL-6*. When incubated with CNP or cANF,^4–23^ the lipopolysaccharide-driven inflammatory profile was reduced significantly. These observations provide clear evidence for a CNP/NPR-C–dependent inhibition of macrophage polarization/activation that otherwise plays a key pathogenic role in AA. This is consistent with the earlier reports of the anti-inflammatory effects of CNP and NPR-C on leukocyte trafficking.^13^ The parallel observations in VSMCs and macrophages reiterate the capacity of CNP to reduce several pathogenic mechanisms in the context of AA and consistent with the notion that CNP mimetics or NPR-C agonists may represent new pharmacological modalities for treating this disorder, in both TAA and AAA.

In sum, we provide persuasive evidence that CNP synthesized and released from endothelial cells and fibroblasts represents a critical innate mechanism that maintains aortic vascular integrity and prevents the development of both TAA and AAA. Further, we establish that NPR-C is the predominant cognate signaling receptor that underpins this cytoprotective activity. Finally, we offer support for the use of CNP-based therapeutics in treating AA, thereby potentially representing an effective pharmacological intervention for this life-threatening disorder.

## Article Information

### Sources of Funding

This work was supported by a program grant (RG/F/23/110123) and a project grant (PG/17/74/33111) from the British Heart Foundation awarded to A.J. Hobbs. S.A. LeMaire is supported, in part, by the Jimmy and Roberta Howell Professorship in Cardiovascular Surgery at Baylor College of Medicine.

### Disclosures

A.J. Hobbs is a scientific advisory board member/consultant for Palatin Technologies, Inc, PharmaIN Corp, and Ascendis Pharma. S.A. LeMaire serves as a consultant for Cerus and has served as a principal investigator for clinical studies sponsored by Terumo Aortic and CytoSorbents. J.C. Tsui has received speaker’s honoraria from Bayer. The other authors have reported no conflicts.

### Supplemental Material

Supplemental Materials and Methods

Table S1

Figures S1–S9

Major Resources Table

ARRIVE Guidelines

References 49–57
